# Correction: Risk of chronic kidney disease in patients with heat injury: A nationwide longitudinal cohort study in Taiwan

**DOI:** 10.1371/journal.pone.0238826

**Published:** 2020-09-02

**Authors:** The PLOS ONE Staff

There is an error in the author byline. Authors Wu-Chien Chien, Chia-Hsien Feng, and Pauling Chu should be noted as contributing equally to this work as corresponding authors. Dr. Chien’s email address is: chienwu@mail.ndmctsgh.edu.tw. Dr. Feng’s email address is: chfeng@kmu.edu.tw. The publisher apologizes for the error.
