# Supplementary material for: Risk of chronic kidney disease in patients with heat injury: A nationwide longitudinal cohort study in Taiwan
Source: PLoS One. 2020 Jul 2;15(7):e0235607. doi: 10.1371/journal.pone.0235607 (PMC7332078; doi:10.1371/journal.pone.0235607)
Supplement: S1 Table — (DOCX) [file pone.0235607.s001.docx]

**S1 Table. Years of follow-up**

| **Heat injury** | **Min** | **Median** | **Max** | **Mean ± SD** |
| --- | --- | --- | --- | --- |
| **With** | 0.01 | 4.11 | 13.18 | 10.40 ± 13.70 |
| **Without** | 0.01 | 9.21 | 13.93 | 10.97 ± 9.84 |
| **Total** | 0.01 | 8.22 | 13.93 | 10.86 ± 10.72 |

SD = Standard deviation; Min = minimum; Max = maximum;

CKD = chronic kidney disease
