# Supplementary material for: Risk of chronic kidney disease in patients with heat injury: A nationwide longitudinal cohort study in Taiwan
Source: PLoS One. 2020 Jul 2;15(7):e0235607. doi: 10.1371/journal.pone.0235607 (PMC7332078; doi:10.1371/journal.pone.0235607)
Supplement: S2 Table — (DOCX) [file pone.0235607.s002.docx]

**S2 Table. Years to CKD**

| **Heat injury** | **Min** | **Median** | **Max** | **Mean ± SD** |
| --- | --- | --- | --- | --- |
| **With** | 0.01 | 3.51 | 11.64 | 4.23 ± 2.87 |
| **Without** | 0.01 | 3.72 | 13.34 | 4.82 ± 4.12 |
| **Total** | 0.01 | 3.88 | 13.34 | 4.62 ± 3.75 |

Min = minimum; Max = maximum; SD = Standard deviation; CKD = chronic kidney disease
