# Supplementary material for: Risk of chronic kidney disease in patients with heat injury: A nationwide longitudinal cohort study in Taiwan
Source: PLoS One. 2020 Jul 2;15(7):e0235607. doi: 10.1371/journal.pone.0235607 (PMC7332078; doi:10.1371/journal.pone.0235607)
Supplement: Attachment — Submitted filename: PONE-D-19-33292_Comments due 28 Dec 2019.docx [file pone.0235607.s003.docx]

PONE-D-19- 33292	Accepted Dec 13 2019

Due Dec 26 2019

Submitted Dec 28 2019

Risk of chronic kidney disease in patients with heat injury: A nationwide longitudinal cohort study in Taiwan

Comments:

The P in P-value is not consistently expressed…. Sometimes P, other time p or even *p*

The authors made no mention of correcting for multiple comparisons.

Indicate the limitations in Discussion with letters or numbers

**Materials and methodsData source**

--space

The diagnostic codes were recorded according to the International Classification of Diseases, Ninth Revision (ICD-9) Reference

**Statistical analysis**

The baseline demographic condition and comorbidities were compared between

the study and control groups using the x2 test for categorical variables and the t-test for

continuous variables [6].

The authors use descriptive statistics in the Results. I suggest they put in about descriptive statistics here.

In supplement Table 1, the median follow-up time of the heat stroke and control

groups were 10.40 ± 13.70 and 10.97 ± 9.84 years, respectively

Median values look more like mean ± standard deviation. Median is usually followed range

A recent study have shown that heat stress induced the increase in plasma…

--- has

**Table1. Characteristics of study in the baseline**

Specify the Location

Tables 1-3

Use a corrective measure for the multiple P-values so that the authors minimize the risk of Type 1 error
