# Supplementary material for: Risk of chronic kidney disease in patients with heat injury: A nationwide longitudinal cohort study in Taiwan
Source: PLoS One. 2020 Jul 2;15(7):e0235607. doi: 10.1371/journal.pone.0235607 (PMC7332078; doi:10.1371/journal.pone.0235607)
Supplement: Attachment — Submitted filename: Response to reviewers.docx [file pone.0235607.s004.docx]

**Response to the reviewers**

**Reviewer #1**

**Comment 1-1**: Was CKD diagnosed according to eGFR? If yes, which equation was used to calculate it (CKD-EPI or other)?

**Reply 1-1**: It had been corrected on page 8, line 6 – 8.

Patients with CKD were divided into subgroups according to their eGFR, which was calculated according to MDRD (modification of diet in renal disease study) equation [8].

**Comment 1-2**: The paper would gain a lot of relevance if the associations were to be performed again according to the various CKD stages (1-5) or at least by subdividing patients in two groups, one for CKD stage 1 to 3a, the other from CKD stage 3b to 5.

**Reply 1-2**: It had been corrected on page 16, line 1 – 8 and page 16, table 5.

Furthermore, the effect of HI on the CKD subgroups were examined according to ICD 9 codes in Table 5. The effect of HI was statistically significant in those CKD stage 2-5 and CKD of unknown stage group. HI patients compared with control group showed increased CKD stage 2 risk (95% CI = 1.038-2.014, adjusted HR: 1.432, P = 0.029), CKD stage 3 risk (95% CI = 4.232-8.496, adjusted HR: 5.265, P < 0.001), CKD stage 4 risk (95% CI = 5.998-11.225, adjusted HR: 7.984, P < 0.001), and CKD stage 5 risk (95% CI = 9.137-20.986, adjusted HR: 11.106, P < 0.001) , respectively.

**Comment 1-3**: In figure 2, there seems to be an acceleration of CKD cases in the last 3 years in the HI group, can you please comment (heat wave in Taiwan or other explanation?)

**Reply 1-3**: It had been explained on page 12, line 10 – 13.

There seems to be an acceleration of CKD cases in the last 3 years in the HI group. It might be because of the annual average temperature was higher and there was more days with daily highest temperature > 35 ^o^C in the last 3 years according to the database of Taiwan Central Weather Bureau.

**Minor comment:**

**Abstract**

**Comment 1-4**: Please correct “newly diagnosed with HI were followed”.

**Reply 1-4**: It had been corrected on page 3, line 10 and 11.

We enrolled patients with HI who were followed in NHIRD system between 2000 and 2013.

**Introduction**

**Comment 1-5**: Heat is repeated three times in “Heat injury (HI) is the accumulation of heat resulting in the body’s inability to tolerate heat.”

**Reply 1-5**: It had been corrected on page 4, line 11.

Heat injury (HI) is the accumulation of heat resulting in the body’s inability to tolerate it.

**Rephrase**

**Comment 1-6**: reductions in nephrons number, insufficient blood supply, cell cycle disruption, and disrupted repair mechanisms, which eventually CAUSE chronic kidney disease (CKD).

**Reply 1-6**: It had been corrected on page 5, line 9.

However, sustained AKI can lead to renal interstitial fibrosis, reductions in nephron number, insufficient blood supply, cell cycle disruption, and disrupted repair mechanisms, which eventually cause chronic kidney disease (CKD).

**Comment 1-7**: Rephrase sentence “In addition to HI, other systemic co-mobidities were also examined in the multivariate analysis model to investigate whether HI is an independent risk factor for CKD, which develops in the majority of the population.”

**Reply 1-7**: It had been rephrased on page 5, line 18 and 19.

In addition to HI, other systemic co-mobidities were also examined in the multivariate analysis model to investigate whether HI is an independent risk factor for CKD. ~~, which develops in the majority of the population.~~

**Comment 1-8**: Separate “Materials and methodsData source”

**Reply 1-8**: It had been separated on page 6, line 3.

**Results**

**Comment 1-9**: separate “andcomorbidity”

**Reply 1-9**: It had been separated on page 10, line 16 and 17.

and comorbidity

**Comment 1-10**: Several word corrections still appear in the text, for example “The risk of CKD is was” and “in the heat stroke and matched groups,” please correct.

**Reply 1-10**: It had been corrected on page 12, line 8 and page15, line 5.

The risk of CKD ~~is~~ was more pronounced and was higher after the fourth year of follow-up in the heat stroke group than in the control group.

We also investigated the risk of ESRD in the heat stroke and matched groups. ~~,~~

**Comment 1-11**: Please correct “Patients with heat stroke had higher CKD events”

**Reply 1-11**: It had been corrected on page 12, line 19.

Patients with heat stroke had higher CKD events than the controls.

**Comment 1-12**: Discussion rephrase (Has and repetition if increase) “A recent study HAS shown that heat stress induced the increase in plasma lipopolysaccharide concentration and significant increases”

**Reply 1-12**: It had been rephrased on page 18, line 19 and 20.

A recent study ~~have~~ has shown that heat stress induced the increase in plasma lipopolysaccharide concentration, ~~and significant increases in~~ anti-inflammatory cytokines (IL-10 and IL-1ra ) and inflammatory responsive cytokines (IL-6)

**Comment 1-13**: “which are in concordance with the findings in previous studies”. Please cite them here

**Reply 1-13:** This sentence had been deleted on page 19, line 7 and 8.

~~Our results demonstrate the higher risk of CKD after heat stroke, which are in concordance with the findings in previous studies.~~

**Response to the reviewer 2:**

**Comment 2-1**: Kindly put in the LINE numbers so it facilitates identification of the lines that the reviewer needs to comment on.

**Reply 2-1**: Line numbers had been put in the manuscript.

**Comment 2-2**: Also, the authors should apply correction procedures for multiple comparisons. This is the major part of my comments as it might impact on the conclusions.

**Reply 2-2**: It had been corrected on page 9, line 18 and 19.

The type I error due to multiple testing was corrected by the Bonferroni method. The P value < 0.001 was considered to be significant for multiple comparisons.

**Comment 2-3**: The P in P-value is not consistently expressed… Sometimes P, other times p or even *p*

**Reply 2-3**: It had been corrected in the manuscript page 9, line 15 and 17; page 15, line 9.

**Comment 2-4**: Materials and methodsData source

--space

**Reply 2-4**: It had been corrected on page 6, line3.

**Comment 2-5**: The authors made no mention of correcting for multiple comparisons.

**Reply 2-5**: It had been corrected on page 9, line 18 and 19.

The type I error due to multiple testing was corrected by the Bonferroni method. The P value < 0.001 was considered to be significant for multiple comparisons.

**Comment 2-6**: Indicate the limitations in Discussion with letters or numbers.

**Reply 2-6**: It had been corrected on page 20, line 11.

The strengths of our retrospective study included the national database derived from ~~1~~ one million sampled cases.

**Materials and methods**

**Comment 2-7**: The diagnostic codes were recorded according to the International Classification of Diseases, Ninth Revision (ICD-9), Reference

**Reply 2-7**: It had been corrected, on page 23, line 6 - 8.

Reference [7] International Classification of Diseases, Ninth Revision (ICD-9), Centers for Disease Control and Prevention, 1998, available from <https://www.cdc.gov/nchs/icd/icd9.htm>

**Statistical analysis**

**Comment 2-8**: The baseline demographic condition and comorbidities were compared between the study and control groups using the x2 test for categorical variables and the t-test for continuous variables [6].

The authors use descriptive statistics in the Results. I suggest they put in about descriptive statistics here.

**Reply 2-8**: It had been corrected, on page 9, line 4.

The baseline demographic condition and comorbidities were compared between the study and control groups using descriptive statistics. Chi-square test were used for categorical variables and *t*-test for continuous variables.

**Comment 2-9**: In supplement Table 1, the median follow-up time of the heat stroke and control groups were 10.40 ± 13.70 and 10.97 ± 9.84 years, respectively

Median values look more like mean ± standard deviations. Median is usually followed range

**Reply 2-9**: It had been corrected, on page 16, line 15.

In Table S1-1 and S1-2, the ~~median~~ mean follow-up time of the heat stroke and control groups were 10.40 ± 13.70 and 10.97 ± 9.84 years, respectively.

**Comment 2-10**: A recent study have shown that heat stress induced the increase in plasma…

--- has

**Reply 2-10**: It had been rephrased on page 18, line 19.

A recent study ~~have~~ has shown that heat stress induced the increase in plasma lipopolysaccharide concentration,

**Comment 2-11**: Table 1. Characteristics of study in the baseline

Specify the Location

**Reply 2-11**: It had been corrected on page 11, table 1.

Location in Taiwan

**Comment 2-12**: Table 1-3. Use a corrective measure for the multiple P-values so that the authors minimize the risk of Type 1 error.

**Reply 2-12**: It had been corrected on page 9, line 18 and 19.

The type I error due to multiple testing was corrected by the Bonferroni method. The P value < 0.001 was considered to be significant for multiple comparisons.
