# Supplementary material for: Risk of chronic kidney disease in patients with heat injury: A nationwide longitudinal cohort study in Taiwan
Source: PLoS One. 2020 Jul 2;15(7):e0235607. doi: 10.1371/journal.pone.0235607 (PMC7332078; doi:10.1371/journal.pone.0235607)
Supplement: Attachment — Submitted filename: response to the reviewer3.docx [file pone.0235607.s005.docx]

Response to the reviewer 3:

**Comment 1**. Authors need to add some additional information about the Taiwan National Health Insurance Research Database. What years does it cover? What specifically does it capture? For example, it is not clear if data prior to 2000 are available for review, but authors selected 2000-2013 as the study period or if the database started in 2000. This is particularly important for understanding the control group who the authors describe as "never having HI"
**Response 1** : **Thanks for the reviewer’s comment. Taiwan launched a single-payer National Health Insurance program on March 1, 1995. The database of this program contains registration files and original claim data for reimbursement. Large computerized databases derived from this system by the National Health Insurance Administration. We use data from 1995 to 1999 as wash-out period to make sure the cases enrolled from 2000 are new cases.**

**Comment 2.** Authors need to discuss the method used for matching (Figure 1 mentions propensity-score matched controls, but this approach is not mentioned anywhere in the methods). Related to the propensity score, authors are encouraged to include the model results used as the basis for propensity score matching as a supplemental table.
**Response 2: Patients and controls were enrolled and propensity-score-matched (1:4) by age, sex, index date, comorbidities, and baseline medications.**

**Comment 3**. Authors mention 95% confidence intervals for incidence, but these are not presented anywhere (line 10, page 9)
**Response 3: This sentence had been deleted.**

**Comment 4**. Authors state in the methods that they used the Bonferroni method for handling multiple comparisons, but never mention it again. Authors should make note of findings that survive the correction (I'm guessing this might be the * in the tables, but that is not specified).
**Response 4: It had been mentioned in the note of table 1.**

**Comment 5**. Table 1 has percentages for categories calculated out of the entire sample, even for the With and Without groups. Such a percentage makes sense for the Total column, but for the With and Without columns, such a percentage is difficult to interpret especially with the 1:4 matching. The percentages should be calculated out of the "n" for that column, so that when looking at the p-value, the reader can directly look at the column percentages to understand the differences (or lack thereof).
**Response 5: The percentage of table 1 had been re-calculated according to the suggestion of reviewer.**

**Comment 6**. Table 1 - the p-values associated with the comorbidities are not correct based on the frequencies provided. When I run chi-square or Fisher's exact tests on the contingency tables, I get highly significant differences between the With and Without groups on the comorbidity variables.
**Response 6: Some of the frequencies in table 1 was misplaced and miscalculated. It had been corrected, with red words and marked.**

Comment 7. Figure 2: authors report the numbers under the figure as No. at risk, but these correspond to the numbers with CKD. The number at risk is more informative. It isn't clear why there are counts of CKD given at 0 years, since all individuals are free of CKD at 0. Also, for the HI group, it is not clear why the numbers under the figure stay at 72 for years 11, 12, 13, while there are jumps in the figure, which should correspond to new cases.
**Response 7: It is because the X-axis was not matched correctly with Y-axis. Figure 2 had been redrawn.**

Comment 8. Table 3: Is model 2 a single model with the various categories of HI used as predictors? The use of "stratified by variables" in the table title makes it unclear exactly what was done. Some of the categories have very low n (and a low number of events) which makes them uninformative. Also, 95% CIs should be included for the Adjusted HRs.
**Response 8: Table 3 had been deleted from the manuscript.**

Minor points:

1. line 15 in Abstract: The authors use "13-year follow-up", but they do not have 13 years of follow-up for all individuals. Authors might consider using "13 year observation period" or something similar.
**Response: It had been corrected on page 3, line 15.**

2. line 17 in Abstract: "higher CKD events" should be "an increased risk of CKD"
**Response: It had been corrected.**

3. line 14 on page 4: "Heat stroke is type of" should be "Heat stroke is a type of"
**Response**: It had been corrected.

4. line 15 on page 4: "accounts to 600" should be "accounts for 600"
**Response: It had been corrected.**

5. lines 11-12 on page 7: authors mention that HI patients that could not be matched were excluded - these aren't reported in Figure 1 anywhere - how many were excluded due to lack of a match?
**Response: This had been mentioned in new figure 1.**

6. line 17 on page 7: though readers can likely figure it out, authors should define "index date"
**Response:** **It had been defined.**

7. line 18 on page 7: "All enrolled patients had been diagnosed with CKD" is confusing, since not everyone had CKD. I'm guessing the authors mean to say something similar to "Individuals were identified as having CKD if they had a diagnosis"
**Response:** It had been rephrased.

Individuals were identified as having CKD if they had a diagnosis should be confirmed…….

8. line 16 on page 8: "or after 31 December 2013" is confusing - do the authors just mean to say "31 December 2013" since that is the end of the observation period?
**Response: It had been corrected.**

or on 31 December 2013.

9. lines 5-6 on page 9: the sentence starting with "Multivariate Cox models" is confusing, since models were not generated simultaneously for the list of variables - those variables were included in all of the models.
**Response: It had been corrected.**

10. lines 10-12 on page 9: the sentence starting with "The odds ratios of CKD" is awkwardly phrased and should be reworded.
**Response: It had been rephrased.**

The odds ratios of CKD incidence rate after HI ~~with and without adjustments for covariates with P < 0.05~~ in the univariate analysis was used for the conditional logistic regression analyses.

11. Figure 1: "nuknown" is an incorrect spelling (box for Exclusion criteria)
**Response: It had been corrected.**

12. line 12 on page 10: Authors mention no difference in insurance premiums, but this information is not presented in Table 1
**Response: It had been deleted.**

~~insurance premiums (in New Taiwan dollar $),~~

13. Note under Table 1: "continue" should be "continuous"
**Response: It had been corrected.**

14. lines 2-9 on page 12: the description of the curves may not be needed, as there are no statistical tests performed to support the various statements. The larger jumps in the later years could be due to smaller numbers of people still at risk.
**Response: The description of the curves had been deleted.**

15. Tables 4-5 do not need to include the results for the overall sample, since that is already provided in Table 2. They also have the exact same title, so authors should come up with more descriptive table titles.
**Response: The overall sample had been deleted. The table titles had been changed.**

16. Tables 4 and 5 also need some clarification - for example, is the row for "With HD" corresponding to a model where the outcome is CKD with HD vs no CKD? What does "Ratio" represent? Also, there are two columns labeled 95% CI - I'm assuming these are the lower limit and the upper limit for the CI, but that should be clarified. The sample sizes in the groups (for example "With HD" or "CKD Stage II") should be given in the tables.
**Response: It had been corrected in table 4 and 5 (changed to table 3 and 4).**

17. Tables S-1 and S-2: "medium" should be "median". Also, how are the max follow-up times greater than 13 years if the study period is from Jan 1, 2000 to Dec 31, 2013?
**Response: It had been corrected in tables S-1 and S-2. The duration is 14 years from Jan 1, 2000 to Dec 31, 2013.**

**(2000, 2001, 2002, 2003, 2004, 2005, 2006, 2007, 2008, 2009, 2010, 2011, 2012, 2013; totally 14 years.)**

18. line 11 on page 16: "duration of onset of HI and CKD" should be "average time between HI and onset of CKD" or something similar and "duration" on the next line should be changed similarly.
**Response: It had been corrected.**

19. last sentence of the results: did the authors do a statistical test to support this statement?
**Response: The last sentence had been deleted.**

20. line 3 on page 17: "was associated with" should be "had a"
**Response: It had been corrected.**

21. line 8 on page 17: "temperatures" should be "temperature"
**Response: It had been corrected.**

22. line 18 on page 17: is the New York study really based on 19.17 million patients?
**Response: The study population in that study included all residents of New York State. The population of New York State was 19.17 million.**

23: line 14 on page 19: "strongly association" should be "strongly associated"

**Response: It had been corrected.**
